# Reinvestigation of the crystal structure of *N*-(4-chloro­benzyl­idene)-2-hy­droxy­aniline: a three-dimensional structure containing O—H⋯N, O—H⋯O and C—H⋯π(arene) hydrogen bonds

**DOI:** 10.1107/S205698901800244X

**Published:** 2018-02-23

**Authors:** Marisiddaiah Girisha, Hemmige S. Yathirajan, Ravindranath S. Rathore, Christopher Glidewell

**Affiliations:** a Department of Studies in Chemistry, University of Mysore, Manasagangotri, Mysuru 570 006, India; b Centre for Biological Sciences (Bioinformatics), School of Earth, Biological and Environmental Sciences, Central University of South Bihar, Patna 800 014, India; c School of Chemistry, University of St Andrews, St Andrews, Fife KY16 9ST, Scotland

**Keywords:** crystal structure, Schiff bases, mol­ecular conformation, hydrogen bonding, supra­molecular assembly

## Abstract

The mol­ecule of the title compound, C_13_H_10_ClNO, (I), which contains an intra­molecular O—H⋯N hydrogen bond, is almost planar: the dihedral angle between the two aryl rings is only 3.31 (9)°. The mol­ecules of (I) are linked into sheets by two C—H⋯π(arene) hydrogen bonds and the sheets are linked into a three-dimensional structure by O—H⋯O hydrogen bonds. Comparisons are made with the structures of a number of related compounds.

## Abstract 2

The mol­ecules of the title compound are almost planar, with an intra­molecular O—H⋯N hydrogen bond, and they are linked into a three-dimensional framework by a combination of one O—H⋯O hydrogen bond and two C—H⋯π(arene) hydrogen bonds.

## Chemical context   

**Figure 1:**
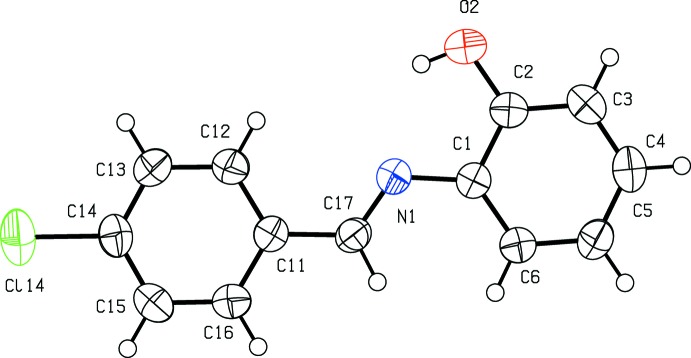
The mol­ecular structure of compound (I), showing the atom-labelling scheme. Displacement ellipsoids are drawn at the 50% probability level.

Schiff bases exhibit a very wide range of biological activities (da Silva *et al.*, 2011 ▸) and are also of inter­est because of the photochromic and thermochromic properties (Hadjoudis & Mavridis, 2004 ▸; Minkin *et al.*, 2011 ▸). The mol­ecular structure of *N*-(4-chloro­benzyl­idene)-2-hy­droxy­aniline (I) was reported in the space group *P*2_1_/*n* a number of years ago [CSD (Groom *et al.*, 2016 ▸) refcode FAKDIE; Kamwaya & Khoo, 1985 ▸]. However, scrutiny of the reported structure reveals a number of unexpected features: the refinement was conducted in a non-standard monoclinic cell having β < 90°; the C—C distances in the aryl rings range between 1.336 and 1.427 Å; no H atoms bonded to C atoms were included; and the C—O—H angle was reported as 88°, which seems very small, while the associated intra­molecular H⋯N distance was only 1.66 Å, which is very short, even for a strong O—H⋯N hydrogen bond. Hence any conclusions drawn from the deposited atomic coordinates may be untrustworthy. The structures of several positional isomers of (I) have been reported recently (Kazak *et al.*, 2004 ▸; Sundararaman *et al.*, 2007 ▸; Saranya *et al.*, 2015 ▸) and in view of these reports and of the widespread applications of Schiff bases, we have accordingly now collected a new data set for compound (I), whose structure we report here (Fig. 1 ▸).

## Structural commentary   

The mol­ecular skeleton of compound (I) (Fig. 1 ▸) is very nearly planar: the r.m.s. deviation from the mean plane through all of the non-H atoms is only 0.043 Å, with a maximum displacement from this plane of 0.0900 (10) Å for atom Cl14. The dihedral angle between the two aryl rings in the mol­ecule of (I) is only 3.31 (9)°. A fairly short intra­molecular O—H⋯N contact (Table 1 ▸) may be an influence on the mol­ecular conformation. The C—C distances within the rings lie in the range 1.377 (3)–1.393 (3) Å for the hy­droxy­lated ring, and 1.366 (3)–1.387 (3) Å for the chlorinated ring, much smaller than the range previously reported (Kamwaya & Khoo, 1985 ▸), while the C—O—H angle is 103.0 (18)°. The inter-axial angle β, as found here and as previously reported, β′, are related by β = (180 - β′) and the atomic coordinates found here can be related to those reported previously, after inversion and a straightforward origin shift, by the transformation (*x*, *y*, −*z*), suggesting that the previous determination may have in advertently used a left-handed axis set.
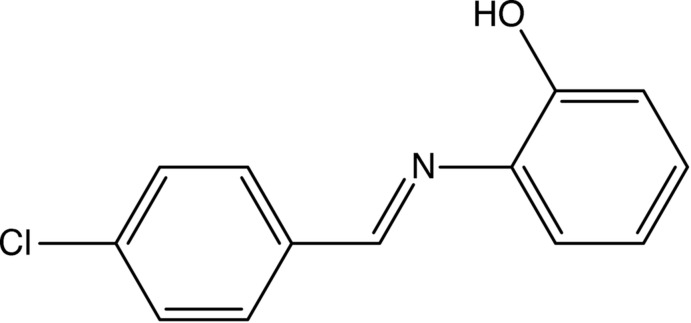


## Supra­molecular features   

**Figure 2:**
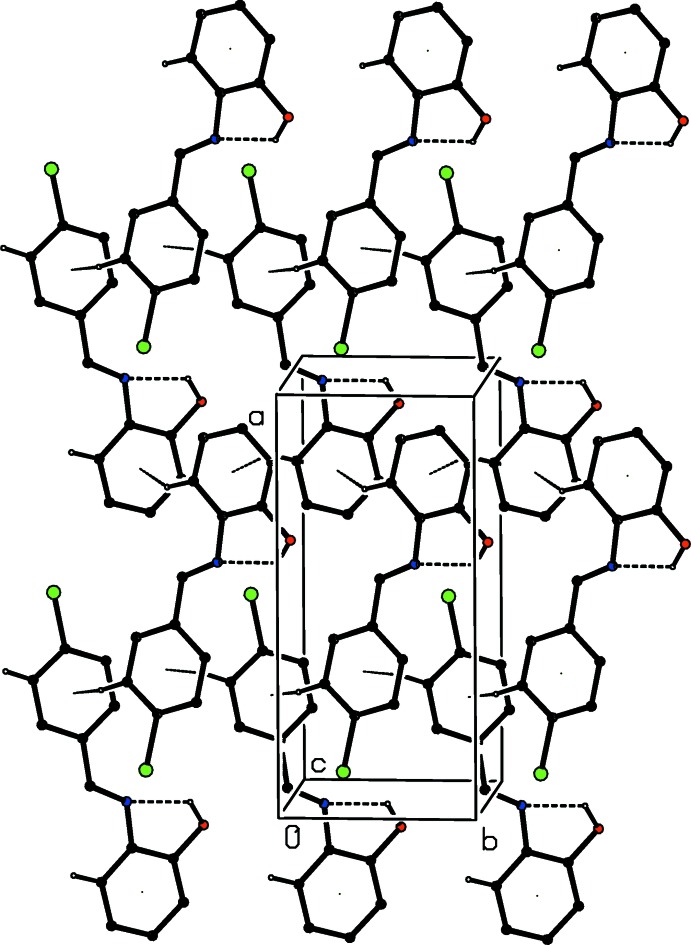
Part of the crystal structure of compound (I), showing the formation of a sheet lying parallel to (001) and built from C—H⋯π(arene) hydrogen bonds. For the sake of clarity, H atoms bonded to C atoms but not involved in the motifs shown have been omitted.

**Figure 3:**
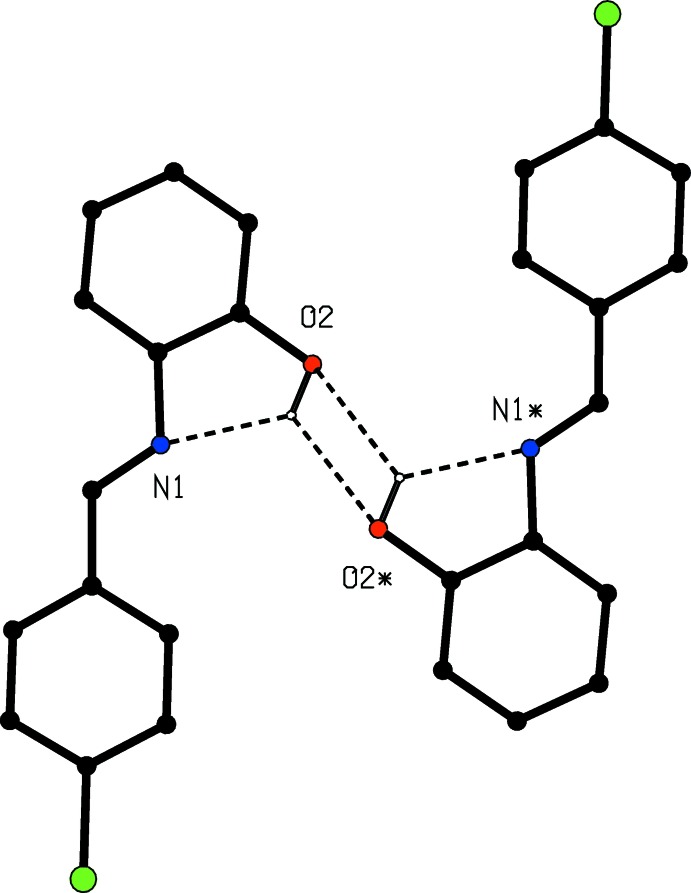
Part of the crystal structure of compound (I), showing the O—H⋯O inter­action between an inversion-related pair of mol­ecules. For the sake of clarity, the unit-cell outline and H atoms bonded to C atoms have been omitted. Atoms marked with an asterisk (*) are at the symmetry position (1 − *x*, 2 − *y*, 1 − *z*).

The supra­molecular assembly is dominated by two C—H⋯π(arene) hydrogen bonds (Table 1 ▸): that having atom C6 as the donor links mol­ecules related by the 2_1_ screw axis along (0.75, *y*, 0.75), and that having atom C15 as the donor links mol­ecules related by the 2_1_ screw axis along (0.25, *y*, 0.75), so forming two distinct types of chain parallel to [010]. In the first of these, the chlorinated ring provides both the donor and the acceptor, while in the second the hy­droxy­lated ring provides both the donor and the acceptor (Fig. 2 ▸). The combination of these two chains links the mol­ecules of (I) into sheets lying parallel to (001) (Fig. 2 ▸). Two sheets of this type, related to one another by inversion, pass through each unit cell, in the domains 0 < *z* < 0.5 and 0.5 < *z* < 1.0. Adjacent sheets are linked into a continuous three-dimensional framework by a combination of a short O—H⋯O contact involving inversion-related pairs of mol­ecules (Fig. 3 ▸), and an aromatic π–π stacking inter­action. The aryl rings (C1–C6) and (C11–C16) in the mol­ecules at (*x*, *y*, *z*) and (1 − *x*, 1 − *y*, 1 − *z*), respectively, which lie in adjacent sheets, make a dihedral angle of 3.31 (9)°: the ring centroid separation is 3.773 (2) Å and the shortest perpendicular distance from the centroid of one ring to the plane of the other is 3.465 (2) Å, giving a ring centroid offset of *ca* 1.49 Å. In the earlier report (FAKDIE; Kamwaya & Khoo, 1985 ▸), the absence of any H atoms bonded to C atoms means that the C—H⋯π(Arene) inter­actions were necessarily overlooked, and the apparent misplacement of the hydroxyl H atom noted above means that the inter­molecular O—H⋯O hydrogen bond was also overlooked.

## Database survey   

**Figure 4:**
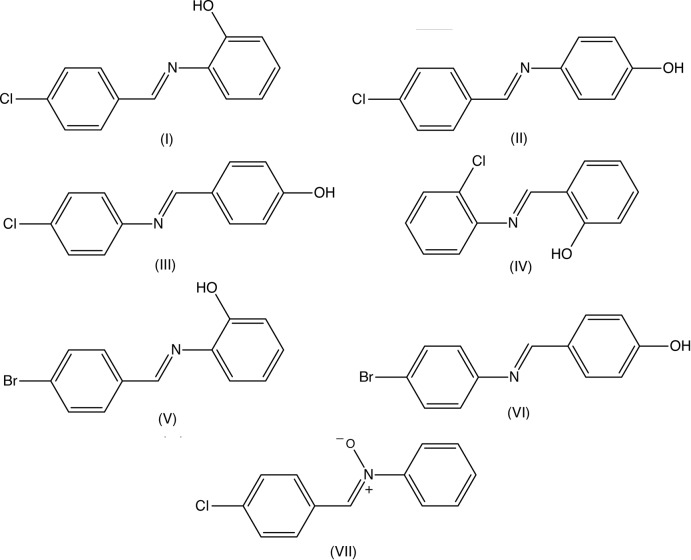
Compound (I) and some closely related analogues.

The structures of a number of Schiff bases which are isomeric with compound (I) have been reported in recent years (see Fig. 4 ▸). In each of compounds (II) (Kazak *et al.*, 2004 ▸) and (III) (Sundararaman *et al.*, 2007 ▸), the mol­ecules are linked by O—H⋯N hydrogen bonds to form chains of the *C*(7) and *C*(8) types, respectively, while in compound (IV) (Saranya *et al.*, 2015 ▸) the sole O—H⋯N inter­action is intra­molecular. The bromo derivative (V) (Jiao *et al.*, 2006 ▸) is isomorphous with the chloro analogue (I), but these two compounds are not strictly isostructural in that the structure of (V) contains only one C—H⋯π(arene) hydrogen bond, as compared with two such bonds in the structure of (I). On the other hand, compounds (III) and (VI) (Jothi *et al.*, 2012 ▸) do appear to be isostructural. Finally, we note the isomeric nitrone (VII), which crystallizes in space group *P* 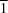 with *Z*′ = 2: each of the two types of mol­ecule forms a *C*(4) chain built from C—H⋯O hydrogen bonds (Vijayalakshmi *et al.*, 2000 ▸).

## Synthesis and crystallization   

To a solution of 2-amino­phenol (0.917 mmol) in ethanol (20 cm^3^), an equimolar qu­antity of 4-chloro­benzaldehyde was added dropwise, with constant stirring, in the presence of a catalytic amount of glacial acetic acid. The mixture was then heated under reflux for 4 h. When the reaction was complete, as judged using thin layer chromatography, the reaction mixture was cooled to ambient temperature and the resulting solid product was collected by filtration and recrystallized from dimethyl sulfoxide, to give crystals suitable for single-crystal X-ray diffraction; m.p. 358 K.

## Refinement   

Crystal data, data collection and structure refinement details are summarized in Table 2 ▸. All H atoms were located in difference maps. The H atoms bonded to C atoms were subsequently treated as riding atoms in geometrically idealized positions with C—H distances of 0.93 Å and with *U*_iso_(H) = 1.2*U*_eq_(C). For the H atom bonded to the O atom, the atomic coordinates were refined with *U*_iso_(H) = 1.5*U*_eq_(O), giving an O—H distance of 0.84 (3) Å. In the final analysis of variance there was a negative value, −3.134, of *K* = [mean(*F_o_* ^2^)/mean(*F*_c_^2^)] for the group of 291 very weak reflections having *F*_c_/*F*_c_(max) in the range 0.000 < *F*_c_/*F*_c_(max) < 0.003: this is probably a statistical artefact.

## Supplementary Material

Crystal structure: contains datablock(s) global, I. DOI: 10.1107/S205698901800244X/zl2725sup1.cif

Structure factors: contains datablock(s) I. DOI: 10.1107/S205698901800244X/zl2725Isup3.hkl

Supporting information file. DOI: 10.1107/S205698901800244X/zl2725Isup3.cml

CCDC reference: 1823227

Additional supporting information:  crystallographic information; 3D view; checkCIF report

## Figures and Tables

**Table 1:** Hydrogen-bond geometry (Å, °) *Cg*1 and *Cg*2 are the centroids of the C1–C6 and C11–C16rings, respectively.

*D*—H⋯*A*	*D*—H	H⋯*A*	*D*⋯*A*	*D*—H⋯*A*
O2—H2⋯N1	0.84 (3)	2.05 (3)	2.626 (2)	125 (2)
O2—H2⋯O2^i^	0.84 (3)	2.44 (3)	2.899 (2)	115 (2)
C6—H6⋯*Cg*1^ii^	0.93	2.79	3.491 (2)	133
C15—H15⋯*Cg*2^iii^	0.93	2.96	3.675 (2)	135

Symmetry codes: (i) 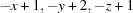; (ii) 
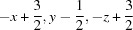
; (iii) 
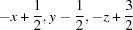
.

**Table 2:** Experimental details

Crystal data	
Chemical formula	C_13_H_10_ClNO
*M*_r_	231.67
Crystal system, space group	Monoclinic, *P*2_1_/*n*
Temperature (K)	296
*a*, *b*, *c* (Å)	13.0830 (17), 5.8746 (6), 14.825 (2)
β (°)	101.521 (4)
*V* (Å^3^)	1116.5 (2)
*Z*	4
Radiation type	Mo *K*α
μ (mm^−1^)	0.32
Crystal size (mm)	0.24 × 0.22 × 0.14

Data collection	
Diffractometer	Bruker APEXII
Absorption correction	Multi-scan (*SADABS*; Bruker, 2012 ▸)
*T*_min_, *T*_max_	0.897, 0.957
No. of measured, independent and observed [*I* > 2σ(*I*)] reflections	11962, 2565, 1556
*R*_int_	0.032
(sin θ/λ)_max_ (Å^−1^)	0.650

Refinement	
*R*[*F* ^2^ > 2σ(*F* ^2^)], *wR*(*F* ^2^), *S*	0.042, 0.106, 1.02
No. of reflections	2565
No. of parameters	148
H-atom treatment	H atoms treated by a mixture of independent and constrained refinement
Δρ_max_, Δρ_min_ (e Å^−3^)	0.16, −0.21

Computer programs: *APEX2* and *SAINT* (Bruker, 2012 ▸), *SHELXT2014* (Sheldrick, 2015*a* ▸), *SHELXL2014* (Sheldrick, 2015*b* ▸) and *PLATON* (Spek, 2009 ▸).

## supplementary crystallographic information

### Crystal data

**Table:**

C_13_H_10_ClNO	*F*(000) = 480
*M**_r_* = 231.67	*D*_x_ = 1.378 Mg m^−^^3^
Monoclinic, *P*2_1_/*n*	Mo *K*α radiation, λ = 0.71073 Å
*a* = 13.0830 (17) Å	Cell parameters from 2571 reflections
*b* = 5.8746 (6) Å	θ = 2.3–27.6°
*c* = 14.825 (2) Å	µ = 0.32 mm^−^^1^
β = 101.521 (4)°	*T* = 296 K
*V* = 1116.5 (2) Å^3^	Block, brown
*Z* = 4	0.24 × 0.22 × 0.14 mm

### Data collection

**Table:**

Bruker APEXII diffractometer	2565 independent reflections
Radiation source: fine focus sealed tube	1556 reflections with *I* > 2σ(*I*)
Graphite monochromator	*R*_int_ = 0.032
φ and ω scans	θ_max_ = 27.5°, θ_min_ = 2.3°
Absorption correction: multi-scan (SADABS; Bruker, 2012)	*h* = −16→16
*T*_min_ = 0.897, *T*_max_ = 0.957	*k* = −7→7
11962 measured reflections	*l* = −18→19

### Refinement

**Table:**

Refinement on *F*^2^	0 restraints
Least-squares matrix: full	Hydrogen site location: mixed
*R*[*F*^2^ > 2σ(*F*^2^)] = 0.042	H atoms treated by a mixture of independent and constrained refinement
*wR*(*F*^2^) = 0.106	*w* = 1/[σ^2^(*F*_o_^2^) + (0.0322*P*)^2^ + 0.4436*P*] where *P* = (*F*_o_^2^ + 2*F*_c_^2^)/3
*S* = 1.02	(Δ/σ)_max_ < 0.001
2565 reflections	Δρ_max_ = 0.16 e Å^−^^3^
148 parameters	Δρ_min_ = −0.21 e Å^−^^3^

### Special details

**Table:**

Geometry. All esds (except the esd in the dihedral angle between two l.s. planes) are estimated using the full covariance matrix. The cell esds are taken into account individually in the estimation of esds in distances, angles and torsion angles; correlations between esds in cell parameters are only used when they are defined by crystal symmetry. An approximate (isotropic) treatment of cell esds is used for estimating esds involving l.s. planes.

### Fractional atomic coordinates and isotropic or equivalent isotropic displacement parameters (Å^2^)

**Table:**

	*x*	*y*	*z*	*U*_iso_*/*U*_eq_	
C1	0.65441 (13)	0.6411 (3)	0.62783 (12)	0.0374 (4)	
C2	0.68202 (15)	0.8448 (3)	0.59096 (13)	0.0423 (5)	
O2	0.60543 (11)	0.9939 (2)	0.55382 (11)	0.0604 (4)	
H2	0.5507 (19)	0.928 (5)	0.5621 (18)	0.091*	
C3	0.78503 (15)	0.8963 (4)	0.59195 (14)	0.0494 (5)	
H3	0.8029	1.0334	0.5678	0.059*	
C4	0.86113 (15)	0.7427 (4)	0.62911 (14)	0.0523 (5)	
H4	0.9307	0.7753	0.6290	0.063*	
C5	0.83542 (15)	0.5410 (4)	0.66645 (14)	0.0500 (5)	
H5	0.8876	0.4392	0.6921	0.060*	
C6	0.73255 (14)	0.4898 (3)	0.66596 (13)	0.0436 (5)	
H6	0.7155	0.3535	0.6912	0.052*	
N1	0.54588 (11)	0.6144 (3)	0.62148 (10)	0.0422 (4)	
C11	0.39586 (13)	0.3955 (3)	0.63857 (12)	0.0390 (4)	
C12	0.32219 (14)	0.5524 (3)	0.59676 (13)	0.0452 (5)	
H12	0.3437	0.6879	0.5740	0.054*	
C13	0.21690 (15)	0.5086 (4)	0.58864 (14)	0.0507 (5)	
H13	0.1675	0.6142	0.5609	0.061*	
C14	0.18611 (14)	0.3071 (4)	0.62218 (14)	0.0479 (5)	
Cl14	0.05422 (4)	0.24857 (13)	0.60947 (5)	0.0861 (3)	
C15	0.25681 (16)	0.1493 (4)	0.66378 (14)	0.0520 (5)	
H15	0.2347	0.0140	0.6863	0.062*	
C16	0.36183 (15)	0.1950 (3)	0.67172 (14)	0.0496 (5)	
H16	0.4106	0.0889	0.6999	0.059*	
C17	0.50747 (14)	0.4332 (3)	0.64610 (13)	0.0429 (5)	
H17	0.5529	0.3169	0.6705	0.052*	

### Atomic displacement parameters (Å^2^)

**Table:**

	*U*^11^	*U*^22^	*U*^33^	*U*^12^	*U*^13^	*U*^23^
C1	0.0388 (9)	0.0405 (10)	0.0329 (10)	0.0007 (8)	0.0071 (8)	−0.0036 (8)
C2	0.0476 (11)	0.0400 (11)	0.0391 (11)	0.0006 (9)	0.0086 (8)	−0.0013 (9)
O2	0.0563 (9)	0.0476 (9)	0.0756 (11)	0.0059 (7)	0.0095 (8)	0.0162 (8)
C3	0.0549 (12)	0.0458 (12)	0.0498 (12)	−0.0093 (10)	0.0160 (10)	−0.0007 (10)
C4	0.0403 (10)	0.0644 (14)	0.0540 (13)	−0.0073 (10)	0.0139 (9)	−0.0068 (11)
C5	0.0405 (11)	0.0565 (13)	0.0525 (13)	0.0062 (10)	0.0081 (9)	0.0010 (11)
C6	0.0430 (10)	0.0431 (11)	0.0448 (11)	0.0009 (9)	0.0085 (8)	0.0024 (9)
N1	0.0386 (8)	0.0434 (9)	0.0440 (10)	0.0007 (7)	0.0068 (7)	0.0020 (8)
C11	0.0388 (10)	0.0414 (11)	0.0364 (10)	0.0008 (8)	0.0064 (8)	−0.0022 (9)
C12	0.0460 (11)	0.0401 (11)	0.0489 (12)	−0.0011 (9)	0.0084 (9)	0.0016 (9)
C13	0.0448 (11)	0.0493 (12)	0.0571 (13)	0.0076 (10)	0.0078 (9)	0.0029 (10)
C14	0.0408 (10)	0.0549 (13)	0.0503 (12)	−0.0055 (9)	0.0149 (9)	−0.0070 (10)
Cl14	0.0454 (3)	0.1019 (5)	0.1145 (6)	−0.0124 (3)	0.0244 (3)	0.0021 (4)
C15	0.0574 (13)	0.0454 (12)	0.0558 (13)	−0.0091 (10)	0.0178 (10)	0.0015 (10)
C16	0.0486 (11)	0.0451 (12)	0.0534 (13)	0.0018 (9)	0.0064 (9)	0.0098 (10)
C17	0.0396 (10)	0.0414 (11)	0.0461 (12)	0.0048 (8)	0.0042 (8)	0.0021 (9)

### Geometric parameters (Å, º)

**Table:**

C1—C6	1.387 (2)	C11—C16	1.384 (3)
C1—C2	1.393 (3)	C11—C12	1.387 (2)
C1—N1	1.413 (2)	C11—C17	1.459 (2)
C2—O2	1.361 (2)	C12—C13	1.383 (3)
C2—C3	1.379 (3)	C12—H12	0.9300
O2—H2	0.84 (3)	C13—C14	1.375 (3)
C3—C4	1.375 (3)	C13—H13	0.9300
C3—H3	0.9300	C14—C15	1.366 (3)
C4—C5	1.377 (3)	C14—Cl14	1.7324 (19)
C4—H4	0.9300	C15—C16	1.382 (3)
C5—C6	1.378 (3)	C15—H15	0.9300
C5—H5	0.9300	C16—H16	0.9300
C6—H6	0.9300	C17—H17	0.9300
N1—C17	1.262 (2)		
			
C6—C1—C2	118.87 (17)	C16—C11—C17	119.35 (17)
C6—C1—N1	127.23 (18)	C12—C11—C17	121.94 (17)
C2—C1—N1	113.90 (16)	C13—C12—C11	120.45 (19)
O2—C2—C3	120.15 (18)	C13—C12—H12	119.8
O2—C2—C1	118.93 (17)	C11—C12—H12	119.8
C3—C2—C1	120.92 (17)	C14—C13—C12	119.16 (18)
C2—O2—H2	103.0 (18)	C14—C13—H13	120.4
C4—C3—C2	119.24 (19)	C12—C13—H13	120.4
C4—C3—H3	120.4	C15—C14—C13	121.77 (18)
C2—C3—H3	120.4	C15—C14—Cl14	118.96 (17)
C3—C4—C5	120.68 (18)	C13—C14—Cl14	119.26 (16)
C3—C4—H4	119.7	C14—C15—C16	118.58 (19)
C5—C4—H4	119.7	C14—C15—H15	120.7
C4—C5—C6	120.16 (19)	C16—C15—H15	120.7
C4—C5—H5	119.9	C15—C16—C11	121.36 (18)
C6—C5—H5	119.9	C15—C16—H16	119.3
C5—C6—C1	120.12 (19)	C11—C16—H16	119.3
C5—C6—H6	119.9	N1—C17—C11	123.79 (17)
C1—C6—H6	119.9	N1—C17—H17	118.1
C17—N1—C1	121.83 (16)	C11—C17—H17	118.1
C16—C11—C12	118.69 (17)		
			
C6—C1—C2—O2	−179.76 (17)	C16—C11—C12—C13	0.1 (3)
N1—C1—C2—O2	0.1 (2)	C17—C11—C12—C13	178.31 (18)
C6—C1—C2—C3	0.1 (3)	C11—C12—C13—C14	−0.3 (3)
N1—C1—C2—C3	179.96 (17)	C12—C13—C14—C15	0.4 (3)
O2—C2—C3—C4	−179.48 (18)	C12—C13—C14—Cl14	−178.35 (15)
C1—C2—C3—C4	0.7 (3)	C13—C14—C15—C16	−0.2 (3)
C2—C3—C4—C5	−1.1 (3)	Cl14—C14—C15—C16	178.52 (16)
C3—C4—C5—C6	0.8 (3)	C14—C15—C16—C11	0.0 (3)
C4—C5—C6—C1	0.0 (3)	C12—C11—C16—C15	0.1 (3)
C2—C1—C6—C5	−0.4 (3)	C17—C11—C16—C15	−178.19 (18)
N1—C1—C6—C5	179.72 (18)	C1—N1—C17—C11	−178.31 (16)
C6—C1—N1—C17	−4.9 (3)	C16—C11—C17—N1	−176.83 (18)
C2—C1—N1—C17	175.25 (17)	C12—C11—C17—N1	5.0 (3)

### Hydrogen-bond geometry (Å, º)

Cg1 and Cg2 are the centroids of the C1–C6 and C11–C16rings, respectively.

**Table:**

*D*—H···*A*	*D*—H	H···*A*	*D*···*A*	*D*—H···*A*
O2—H2···N1	0.84 (3)	2.05 (3)	2.626 (2)	125 (2)
O2—H2···O2^i^	0.84 (3)	2.44 (3)	2.899 (2)	115 (2)
C6—H6···*Cg*1^ii^	0.93	2.79	3.491 (2)	133
C15—H15···*Cg*2^iii^	0.93	2.96	3.675 (2)	135

Symmetry codes: (i) −*x*+1, −*y*+2, −*z*+1; (ii) −*x*+3/2, *y*−1/2, −*z*+3/2; (iii) −*x*+1/2, *y*−1/2, −*z*+3/2.
